# Self-Powered Wireless Carbohydrate/Oxygen Sensitive Biodevice Based on Radio Signal Transmission

**DOI:** 10.1371/journal.pone.0109104

**Published:** 2014-10-13

**Authors:** Magnus Falk, Miguel Alcalde, Philip N. Bartlett, Antonio L. De Lacey, Lo Gorton, Cristina Gutierrez-Sanchez, Raoudha Haddad, Jeremy Kilburn, Dónal Leech, Roland Ludwig, Edmond Magner, Diana M. Mate, Peter Ó. Conghaile, Roberto Ortiz, Marcos Pita, Sascha Pöller, Tautgirdas Ruzgas, Urszula Salaj-Kosla, Wolfgang Schuhmann, Fredrik Sebelius, Minling Shao, Leonard Stoica, Cristoph Sygmund, Jonas Tilly, Miguel D. Toscano, Jeevanthi Vivekananthan, Emma Wright, Sergey Shleev

**Affiliations:** 1 Biomedical Sciences, Faculty of Health and Society, Malmö University, Malmö, Sweden; 2 Institute of Catalysis and Petrochemistry, Madrid, Spain; 3 Chemistry, Faculty of Natural and Environmental Sciences, University of Southampton, Southampton, United Kingdom; 4 Analytical Chemistry/Biochemistry and Structural Biology, Lund University, Lund, Sweden; 5 Analytische Chemie, Ruhr-Universität Bochum, Bochum, Germany; 6 School of Biological and Chemical Sciences, University of London, London, United Kingdom; 7 School of Chemistry, National University of Ireland Galway, Galway, Ireland; 8 Food Science & Technology, BOKU-University of Natural Resources and Life Sciences, Vienna, Austria; 9 Chemical and Environmental Sciences, University of Limerick, Limerick, Ireland; 10 Novosense AB, Lund, Sweden; 11 Novozymes A/S, Bagsværd, Denmark; Texas A&M University, United States of America

## Abstract

Here for the first time, we detail self-contained (wireless and self-powered) biodevices with wireless signal transmission. Specifically, we demonstrate the operation of self-sustained carbohydrate and oxygen sensitive biodevices, consisting of a wireless electronic unit, radio transmitter and separate sensing bioelectrodes, supplied with electrical energy from a combined multi-enzyme fuel cell generating sufficient current at required voltage to power the electronics. A carbohydrate/oxygen enzymatic fuel cell was assembled by comparing the performance of a range of different bioelectrodes followed by selection of the most suitable, stable combination. Carbohydrates (viz. lactose for the demonstration) and oxygen were also chosen as bioanalytes, being important biomarkers, to demonstrate the operation of the self-contained biosensing device, employing enzyme-modified bioelectrodes to enable the actual sensing. A wireless electronic unit, consisting of a micropotentiostat, an energy harvesting module (voltage amplifier together with a capacitor), and a radio microchip, were designed to enable the biofuel cell to be used as a power supply for managing the sensing devices and for wireless data transmission. The electronic system used required current and voltages greater than 44 µA and 0.57 V, respectively to operate; which the biofuel cell was capable of providing, when placed in a carbohydrate and oxygen containing buffer. In addition, a USB based receiver and computer software were employed for proof-of concept tests of the developed biodevices. Operation of bench-top prototypes was demonstrated in buffers containing different concentrations of the analytes, showcasing that the variation in response of both carbohydrate and oxygen biosensors could be monitored wirelessly in real-time as analyte concentrations in buffers were changed, using only an enzymatic fuel cell as a power supply.

## Introduction

Self-contained, i.e., wireless and self-powered, bioelectronic devices are of major scientific and practical importance with potential applications as self-sustaining implantable medical devices, and in environmental, and biocomputing applications. Implantable wireless sensor-systems allow for localised real-time biomedical monitoring of analyte molecules of interest, *e.g*. carbohydrates, oxygen, neurotransmitters etc, and can enable the combination of sensor readings with treatment. Such capabilities can bring major healthcare benefits by increasing early detection of emergency conditions and diseases in at-risk patients and/or by providing a wide range of healthcare services for people with various degrees of cognitive and physical disabilities. Development of implantable self-contained biodevices is an interdisciplinary research field spanning scientific, computing, engineering, and medical disciplines, with significant research focus and a rapid growth in the number of publications in the area of bioelectronics [1]–[8].

An implantable biodevice requires a power source, which ideally should not be an enclosed battery, since this restricts the possibility for miniaturisation, as well as limits the lifetime, considering the fact that a battery eventually would need to be exchanged. An implantable device should rather be self-powered. Power can potentially be extracted from the human body in a variety of ways, including mechanical energy in the form of body movements, hydraulic energy in the form of blood flow, or chemical energy released from compounds present at the implant location. Alternatively, power could be supplied wirelessly *via* radio frequency (RF) power circuits by an externally located source.

Several prototypes of different implantable medical devices have been described utilising a range of methods of extracting and delivering power. To highlight some examples, Amsel et al. recently designed a prototype self-powered light therapeutic device to be implanted inside a blood vessel in order to perform blood irradiation therapy, drawing power *via* the hydraulic energy in the blood flow [9]. Borton et al. designed and implanted a wireless neural recording device housed in a titanium enclosure [10]. The device was powered by a Li-ion battery, which could be recharged *via* an inductive transcutaneous power link. Furthermore, an implantable blood flow sensor was described by Cheong et al., where the wireless sensor was powered through an inductive link [11]. In this paper we have chosen to employ a biological fuel cell (BFC) as power supply, converting chemical energy available in carbohydrates, readily available in the human body, into electrical energy.

Several BFCs implanted into different living organisms have been reported in the literature, with many developments in recent years [12]–[21]. One example of an enzyme based BFC was implanted into a living lobster, and energy harvested from sugar and oxygen available inside the body was used to power small electrical devices [18]. By incorporating a charge pump and DC-DC converter, the voltage of the BFC was sufficient, when placed in a fluidic system with human serum spiked with glucose, to power a pacemaker [22].

By employing enzymes as catalysts, enzymatic FCs (EFCs) can efficiently oxidise a wide variety of fuels and reduce a range of oxidants, usually abundantly available oxygen. Enzyme based FCs are very promising when considering biocompatibility, selectivity, efficiency, and sensitivity, and they have many potential applications for powering nano- and micro-electronic portable devices, drug delivery systems, biosensors, and implantable biomedical devices [3], [23]–[25]. By utilising appropriate enzymes, EFCs can generate energy from carbohydrates and oxygen that are readily available at sites of implantation. The theoretical performance limits of EFCs depend on the particular biofuel/biooxidant couple, although the parameters of EFCs achieved so far are still far from the thermodynamic limits. For a glucose/oxygen EFC operating *in vitro*, where both biofuel and biooxidant are also readily available *in vivo*, with monolayer coverage on a planar surface the calculated limits are a current density of a few hundred µA cm^−2^ and a maximum cell voltage of roughly 1.2 V. In practice, the obtained voltage of reported EFCs is significantly lower due to the large overpotential needed for oxidation of biofuels and reduction of the biooxidant, often with a maximum power output well below an operating cell voltage of 0.5 V. The current output is also often less than the theoretical limit due to, *e.g.*, insufficient loading of enzymes at the electrode surfaces, non-optimal efficiency in the electronic coupling between the enzyme and the electrode, facilitated either directly (*i.e.*, direct electron transfer, DET) or using mediators (*i.e.*, mediated electron transfer, MET), and diffusion restrictions [24], [25]. A major challenge is to match the output of the EFC with the input requirement of electronic components in the biodevice, as modern semiconductor based circuits require 0.4–0.5 V as a minimum voltage [26], while providing sufficient current. To be able to harvest energy from the body with an EFC and use it to power a completely self-contained, *i.e.*, self-powered and wireless, biodevice, the voltage needs to be at a level high enough for electronics to be operated. A challenge in designing such self-powered biodevices is therefore to construct a suitable EFC based power supply module.

The concept of utilising BFCs to create self-powered electrochemical biosensing devices has been explored in the literature [21], [27]–[30], and was also recently reviewed [31]. However, to the best of our knowledge, no reports have previously demonstrated the operation of a fully self-sustained biodevice with separate sensing bioelectrodes and wireless radio signal transmission, all powered by a BFC. In this paper, we report on the design and demonstration of the operation of autonomous sensing biodevices, which were realised by implementing bioelectrodes for sensing, an energy harvesting module (voltage amplifier together with capacitor), and a radio transmitter, with the power demands supplied by an EFC, generating a sufficient current at the required voltage to power all electronics. Electric power generation was achieved by employing enzyme based carbohydrate oxidising and oxygen reducing electrodes, thereby creating a carbohydrate/oxygen EFC. The operation of bioelectrodes for power generation was optimised by investigating a large number of different designs, selecting the most suitable biodevices in order to create a combined multi-enzyme FC. Sensing was realised by choosing suitable enzyme based electrodes employed as biosensors towards carbohydrates and molecular oxygen, as both oxygen and carbohydrates (*viz.*, glucose) are of interest in biomedical sensing, to demonstrate the operation of the self-contained biodevice. Furthermore, in order to utilise the BFC as a power supply for sensors, a suitable power supply and electronic unit together with computer control software was designed and combined with a standard receiving USB unit to create wireless self-powered sensing biodevices. The operation of the developed devices was investigated in buffers containing a range of concentrations of oxygen and carbohydrates, simultaneously drawing power from the same solution.

## Materials and Methods

### Chemicals and equipment

All chemicals used were of analytical grade. For biodevice testing, an acetate buffer (100 mM, pH 4.5) and Tris-HCl buffer (100 mM, pH 7.4) containing 50 mM CaCl_2_ were used as catholyte and anolyte, respectively. Electrochemical characterization was performed using a µAutolab Type III/FRA2 potentiostat/galvanostat from Metrohm. The operating voltages of BFCs were additionally monitored using a 72–7740 multimeter from Tenma Corporation. Oxygen and air saturated buffers were obtained by continuously bubbling the solutions with oxygen and air.

### Bioelectrodes

A range of designs of bioanodes and biocathodes were combined to enable demonstration of a complete working prototype biodevice, together with suitable carbohydrate and oxygen biosensors. All of these individual bioelectrodes have been detailed previously, and are thus only briefly described herein, for full details the reader is referred to the relevant literature citation in each case.

### Carbohydrate oxidising bioanodes

Three different DET and MET bioanodes based on cellobiose dehydrogenase (CDH) enzyme electrodes were utilised in the biodevice test. Firstly, three-dimensional (3D) gold electrodes modified with gold nanoparticles (AuNPs) were functionalised with a mixture of thiols, onto which *Corynascus thermophilus* CDH (*Ct*CDH) was cross-linked on the surface of the electrode using glutaraldehyde as cross-linker through the formation of imine bonds [32], [33]. Secondly, 3D microstructured carbon electrodes were utilised as support for deposition of diazonium salt activated single-walled carbon nanotubes (SWCNTs) onto which *Ct*CDH was covalently attached, similarly to the approach reported previously using *Phanerochaete sordida* (*Ps*CDH) [34]. Thirdly, *Ct*CDH was directly adsorbed onto diazonium salt activated three-dimensional (3D) hierarchical carbon electrodes, in a similar manner as SWCNTs modified glassy carbon electrodes [34]. Finally, a MET design was utilised by employing a redox hydrogel based on entrapment of *Myrococcum thermophilum* CDH (*Mt*CDH) within a two electron acceptor toluidine blue redox polymer on graphite electrodes [35], [36].

### Oxygen reducing biocathodes

As for the bioanodes, both DET and MET approaches were employed in the design of different biocathodes, based on oxygen reduction by extensively investigated blue multicopper oxidases (BMCOs), *viz.*, laccase (Lc) and bilirubin oxidase (BOx) [37]. Firstly, 3D hierarchical carbon electrodes were used as a supporting platform for immobilisation of *Trametes hirsuta* Lc (*Th*Lc) [38]. By attaching the Lc covalently to aminophenyl modified electrodes *via* imino-bond formation a favourable orientation of the enzyme is achieved. In addition, the high surface area of the 3D electrode allows a high coverage of enzyme on the electrode and its CNT network enhances the rate of DET reactions. Secondly, *Th*Lc was covalently attached to low density graphite electrodes modified with AuNPs using diazonium salt electrochemical reduction [39]. AuNPs were covalently linked to the functionalized electrode, resulting in a large coverage of AuNPs within the porous structure of the graphite. The porous surface of the low density graphite electrode allows a high coverage of enzyme on the electrode and the monolayer of AuNPs may enhance the rate of DET reactions. Thirdly, covalent immobilisation of *Th*Lc onto 3D hierarchical carbon nanotube electrodes *via* modification with anthraquinone derivatives was utilised to provide an additional type of biocathode [40]. As in the two above examples, proper orientation of the enzyme results in efficient DET based bioelectrocatalytic reduction of oxygen. Finally, two further biocathode designs employing *Myrothecium verrucaria* bilirubin oxidase (*Mv*BOx) were utilised. Nanoporous gold electrodes were modified with enzyme and coated with a specifically designed electrodeposition polymer layer resulting in a DET biocathode showing a stable faradaic response to oxygen and only slight inhibition by fluoride ions [41]. A MET based biocathode was designed by co-immobilising *Mv*BOx within a [Os(2,2′-bipyridine)_2_(polyvinylimidazole)Cl]^+^ redox hydrogel on glassy carbon electrodes, modified with multi-walled CNTs, using a diepoxide crosslinking agent, similarly as previously outlined, but with glassy carbon instead of graphite electrodes and diepoxide instead of glutaraldehyde crosslinking [42].

### Carbohydrate and oxygen sensitive electrodes

A carbohydrate (glucose, lactose, cellobiose, *etc*) sensitive biosensor was constructed using *Ct*CDH adsorbed onto screen printed electrodes and stabilized using glutaraldehyde as crosslinker [43], [44]. The biosensor has a wide linear range and high selectivity for carbohydrates, enabling operation in physiological fluids. This sensor was utilised as the carbohydrate sensitive bioelement during the biodevice testing.

The oxygen biosensor was fabricated by oriented immobilisation of a newly developed blood-tolerant Lc onto AuNP modified gold electrodes, following a previously developed protocol for *Mv*BOx [45]. This newly evolved Lc was generated by directed evolution and showed activity for oxygen reduction under neutral pH conditions, and indeed retained this activity in actual blood samples [46].

## Results and Discussion

### Design of electronic components

To address differences of voltage and power production and requirements between the electronics and EFCs, an energy-harvesting electronics module was developed. One of the tasks for the energy-harvesting module is to amplify the low cell voltage that the EFC delivers to provide a usable voltage level for standard electronic components. In addition, even state-of-the-art commercially available low power electronics require significant energy for operation, *e.g.*, 135 mW for radio transmission and 3–30 mW for sampling [47]. Thus, the energy-harvesting module was also designed to store energy and to control activation of the electronics.

**Figure 1:**
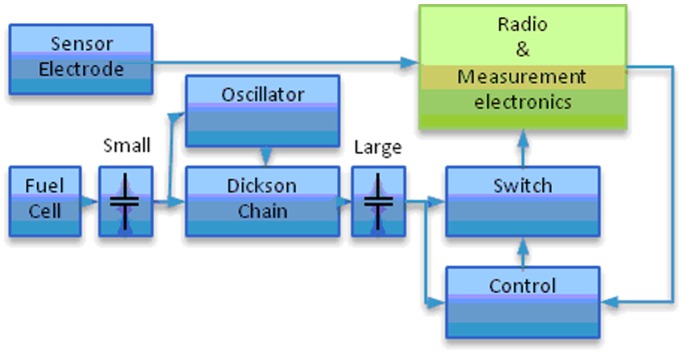
Charge pump design. Overall scheme of the charge pump design divided into different modules connected to electronics for sensing, sampling, and wireless radio transmission of data.

The overall hardware design is depicted in Fig. 1, and a more detailed schematic of the power supply unit is presented in Fig. S1. A photograph of the designed electronic unit containing a CC 2530 radio prototype with integrated antenna and micropotentiostat can also be found in Fig. S2. In this unit, electrical energy provided by the EFC is first intermediately stored in a small capacitor (Fig. 1 and C17, Fig. S1) that will subsequently generate a higher temporal current. The next circuit block consists of an oscillator circuit (A1) that drives a Dickson chain (D1 and D2), which steps up the voltage to enable charging of a larger capacitor (four capacitors, C9–C12). When a high enough voltage (approx. 3.8 V) and charge is accumulated in the large capacitor a control module (A2) turns on a high-performance switch (A3), which permits the circuit to provide power (output A4) to the radio and measurement electronics for 5 ms, thus draining the large capacitor. When the radio has transmitted the information packet it turns off the switch (A3) and the charging of the large capacitor recommences. The sensed data, during operation of the radio and measurement electronics, is directly transmitted to a remote USB receiver (Fig. S3), which is turned on at all times. When the wireless electronic unit was submerged in saline water containing 150 mM of NaCl it displayed a packet error ratio as low as 1% (basically the same as in air) and a range of 5 m for radio signal transmission.

To integrate the sensing unit with the data transmission system a simple potentiostat was incorporated into the electronic device. One of the BFC electrodes serves as a counter and reference electrode. A crucial problem with using a micropotentiostat circuit with the self-powered radio system is the long settling time of the micropotentiostat, and the electronics is only turned on for 4 ms to preserve energy. The sensor electrode was therefore constantly loaded by an adjustable 1 MΩ external resistor (R33, Fig. S1) and the measurement amplifiers (O1 and O2, Fig. S1) only turned on momentarily to conduct the measurement. For this purpose the sensor (glucose or oxygen sensitive) was connected to input (B1, Fig. S1) and through the resistor R33 and input B2 to one of the electrodes of the biofuel cell (biocathode or bioanode, respectively) used for electric power generation purpose. In general, the only difference between biosensor electrodes and biofuel cell electrodes was their size. Specifically, microscale electrodes were used to construct biosensors in order to achieve bioelectriocatalytic currents less than 1 µA since the biosensor electronics was designed for maximum current of that value. The sensor electrodes thus generate low currents, which are transformed into voltages, amplified (by two operational amplifiers, O1 and O2, Fig. S1), and thereafter sent to a 12-bit A/D converter (Fig. S4). To minimise errors during the measurements, digital and analogue integration of the sensor signal were used and optimised. Thus, the errors did not exceed 10% of the mean value from the bioanalyte signal. Less than 10% error includes possible change of the signal due to all available variables during measurements attributed to the operational and long term stabilities of sensors, electronics fluctuations, etc. In other words, if the electronic signal attributed to a certain concentration of a bioanalyte (for instance, 1.2 mM of oxygen, *vide infra*) is 1000 units at the beginning of the test period, when re-measured at the end of the test period (*e.g.* 30 min later), the signal for the same concentration of the bioanalyte is found to deviate at most ±99 units from the initial 1000 units signal. The oxygen and carbohydrate sensitive setups were designed in similar ways, but on separate design platforms. The sensor electronics only need one sensing electrode and use the same cathode/anode as the EFC. By continuously loading the sensor electrode (on R33, Fig. S1), a build up of charge is avoided, and the current is proportional to the analyte concentration.

**Figure 2:**
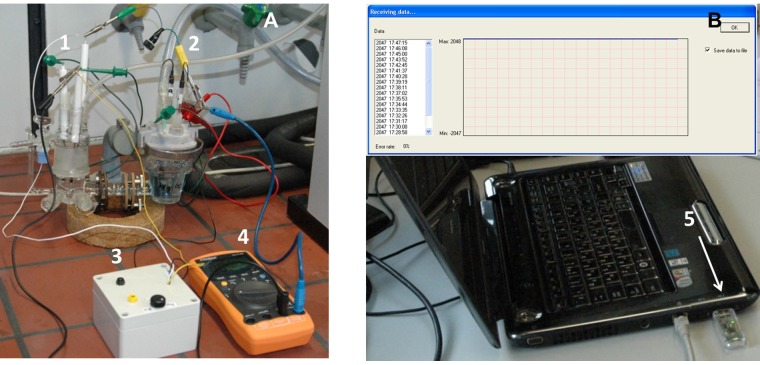
Bench-top device test. Photographs of the set-up for the bench-top device test, showing (A) the oxygen sensitive wireless self-powered biodevice, *i.e.* an EFC (electrochemical cell containing the anodes, 1, and cathodes, 2) connected to the wireless operational unit (white box, 3) and a control device (voltmeter, 4) and (B) a computer with the developed control software and receiver (CC2530 radio highlighted with the white arrow, 5), placed roughly 4 m from the device.

The embedded software to control the designed sensor system was also developed. The main purpose of the software was to control start-up of each module, sample sensor data, and transmit the data to a receiving unit. When sufficient electric energy is harvested, the energy harvesting module powers the radio module to enable data to be measured and sampled. A paired connection to a receiving radio base is established allowing the electronic circuit to send the measured data *via* the radio. After completion of a transmitted message the energy harvesting module is signalled to turn off the power supply and begins to store energy again. To minimise the power used by the radio this process should be as fast as possible; a total duration of 4.4 ms was achieved, of which 3 ms was with the radio transmitter activated (Fig. S5). The transmitted package is sent unsynchronised to the receiver to preserve energy and keep the sensor uptime low. Thus, the developed PC software for the receiver has to operate continuously and wait for the next transmitted package from the receiver (Fig. 2B and Fig. S6). However, this does not present a difficulty as the receiver is connected to a computer and does not consume more than 40 mA. When a data package is received by the receiving radio the package is sent to a USB module, which is connected to the computer (Fig. S3).

The embedded software was optimised to save power and thus allow the sensor to sample more often. As a result the mode of operation is very close to the limits of the radio system, *i.e.*, the radio needs to start-up the main crystal, sample with the ADC, transmit the message, *etc.* The achieved sampling time of 1–3 minute intervals is satisfactory for many types of measurements, *e.g.*, glucose concentration in the human body is not subjectd to such rapid changes over this time interval [48]. It would be possible to increase sampling frequency if the EFC could provide a higher voltage, or if the energy-harvesting module could be improved in efficiency. In total, a minimum requirement of 0.57 V and 44 µA from the EFC is necessary to power the design electronic modules.

### Device demonstration

Prior to actual device testing, the electronic system was calibrated and characterised using a battery power supply instead of an EFC (Figs. S7 and S8). This test system displayed stable and reliable wirelessly monitored signals both for the carbohydrate and oxygen unit, with a low background signal, as described in detail in SI. Calibration and characterisation of the biosensors were not performed herein, as it has already been presented in the published reports detailing the individual biosensors.

The actual device testing of two self-contained biodevices, *viz.*, carbohydrate and oxygen sensitive wireless self-powered biodevices, utilised a combination of the above detailed bioelectrodes as a power source to demonstrate proof-of-principle of the self-sustained biodevices. This was necessary mainly in order to achieve both sufficient current and voltage to power the electronics, *viz.*, ≥ 44 µA and ≥ 0.57 V. The individual current output and OCV of the different bioelectrodes is tabulated in Table S1, and described in detail in the respective references. In general, biocathodes based on *Th*Lc demonstrated high operational potential, as well as high current densities, ultimately limited by diffusion of oxygen toward electrode surfaces, whereas bioanodes limited the overall power generated by the EFC due to the comparatively inefficient utilisation of biofuels by the CDH based bioanodes [7]. In order to maximise the power generated by the EFC, while restricting the amount of material needed for device testing, a two compartment set-up was utilised enabling operation of anode and cathode under different but optimal electrolyte conditions (Fig. 2A), as described below.

For the anolyte, a neutral pH buffer containing calcium ions, known to increase the performance of CDH based electrodes [49], was employed, with lactose present at high concentrations as the biofuel. Since lactose was required to produce power, the carbohydrate concentration could not be significantly decreased during the tests. The lactose concentration in the system was therefore varied from 10 mM up to 100 mM (*vide infra*), while registering the response of the sugar sensing electrode. Glucose was not utilised as fuel in order to maximise the efficiency of the bioanodes, due to the relatively inefficient oxidation of monosaccharides by CDH [50].

For the catholyte, a more acidic pH of 4.5 was used to increase the performance of the Lc based biocathodes, since the enzyme has maximum activity under these conditions [37]. Considering that oxygen was required to produce electrical power the oxygen concentration was varied from 0.25 mM up to 1.2 mM (air saturated up to oxygen saturated solution) [51], while registering the response of the oxygen sensing electrode (*vide infra*). In this way, by combining three MET based bioanodes using *Ct*CDH entrapped within a toluidine-modified redox polymer on graphite and two DET based *Ct*CDH bioanodes on aryl diazonium activated SWCNT-modified nanostructured carbon with two DET biocathodes based on *Th*Lc on CNT/CMF, the multi-enzyme FC was optimised to provide sufficiently high voltage and current, while utilising a small amount of materials (Table S1). By combining these electrodes, an open circuit voltage (OCV) of 0.73 V with a current production of 58 µA at 0.67 V operating voltage could be achieved. The five combined bioanodes were required versus only two biocathodes since the biocathodes produce roughly five times higher current density than the individual bioanodes. The specific bioelectrodes were chosen because they provided the highest current output at the required voltage under the experimental conditions. The use of a BOx based biocathode was foregone, since the Lc based cathodes produce current at higher potentials, and are more efficient at doing so under the chosen test conditions (*i.e.* pH 4.5). The obtained output was sufficient to power the electronics module and demonstrate the proof-of principle of the wireless self-powered biodevices based on radio signal transmission.

The full set-up for the oxygen sensitive biodevice testing, including computer with control software and receiver utilising an EFC as power supply, is shown in Fig. 2. The carbohydrate sensitive biodevice was set-up in a similar way using the same EFC to power the device. In principle, a single compartment set-up operating under physiological conditions could be utilised, but due to the decreased performance of the biocathodes under these conditions, a significantly higher amount of material would be required to fulfil the power requirements of the electronic device.

**Figure 3:**
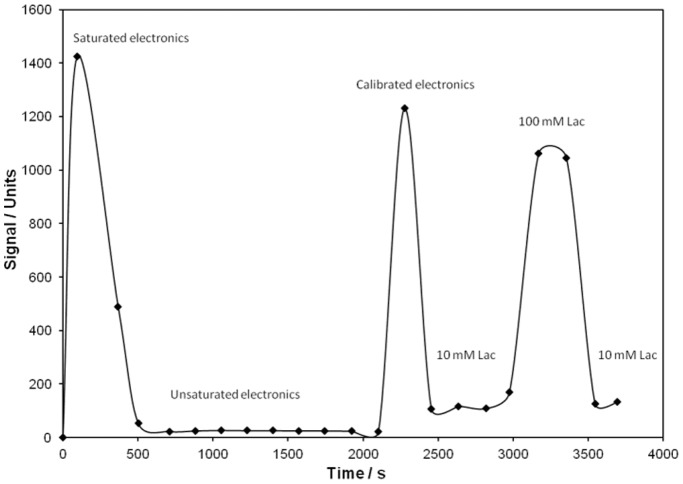
Wireless carbohydrate sensing. Recorded signal from the carbohydrate sensitive self-contained biodevice in buffers with varying lactose concentrations.

In order to demonstrate the operation of the carbohydrate sensitive device, a lactose sensitive enzyme electrode was introduced into the anolyte and connected together with the EFC to the electronics module, while the operating voltage in the system was monitored by an external voltmeter. The recorded response is shown in Fig. 3. First, the procedure to calibrate the system was utilised. When a 100 mM lactose solution was used, the signal of the electronics was adjusted to its highest value, however, avoiding saturation of the electronics (from saturated to unsaturated and then calibrated solution in Fig. 3). After calibration the lactose concentration was decreased to 10 mM, resulting in a drop in the wirelessly measured signal down to *ca.* 120 units. When 100 mM lactose solution was re-introduced into the system, the signal rose to *ca.* 1050 units. Decreasing the lactose concentration to 10 mM once more resulted in a wireless signal of 120 units. This test demonstrates that wireless radio signals from the carbohydrate sensitive biodevice, powered by the EFC, could be measured successfully for a duration of 1 h, during which 22 packages of information were received wirelessly by the receiver located 4 m away.

**Figure 4:**
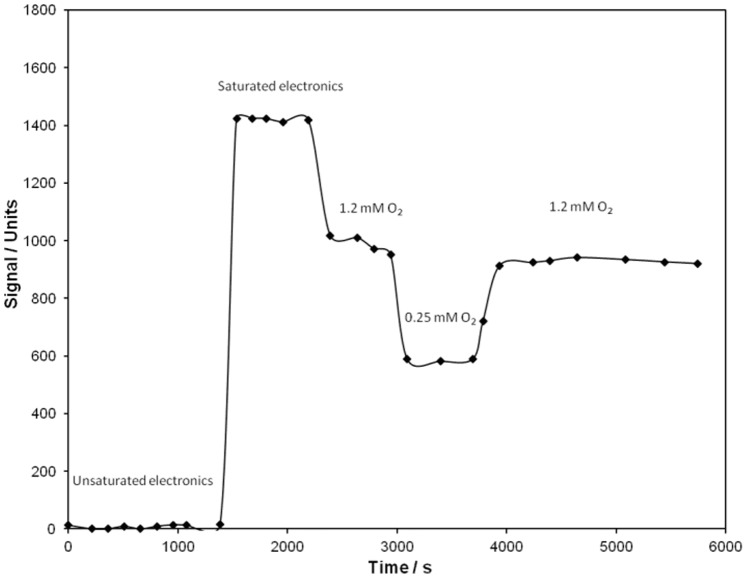
Wireless oxygen sensing. Recorded signal from the self-contained biodevice for oxygen monitoring in buffers with varying oxygen concentrations.

The demonstration of the oxygen sensitive device was performed in a similar manner to that of the carbohydrate sensitive device, utilising the same EFC as power supply, but introducing the oxygen sensitive electrode into the catholyte with the recorded response displayed in Fig. 4. The system was calibrated by saturating the solution with oxygen (1.2 mM O_2_), adjusting the electronics to avoid saturation (setting the response to around 1000 units, Fig. 4). After calibration of the response unit the oxygen level in the catholyte was decreased to 0.25 mM (air saturated solution), which resulted in a reduction of the wirelessly measured radio signal to *ca.* 580 units. When the catholyte was re-saturated with oxygen, the signal increased to roughly 940 units. Wireless signals from the oxygen sensitive biodevice powered by the EFC were measured successfully for 1.5 h, during which time 29 packages of information were received wirelessly by the receiver. The EFC hence required an average time of roughly 3 min to power up the radio module to enable data to be measured and sampled, close to the minimum required sampling time of 1–3 minute intervals for the electronics. The EFC was thus able to efficiently supply energy for all electrical components during the whole duration of the experiment.

The described tests demonstrate that the bench-top prototype for wireless monitoring of oxygen and carbohydrate operated well and could be powered by an EFC. For the duration of the tests, the voltage of the EFC decreased only slightly but not below the critical level required to power the electronics. Evidently, miniaturisation of electronics and optimisation of the EFC power source is needed to move towards more feasible implantable self-powered wireless biodevices. However, the proof-of-principle tests show the possibility to employ miniature self-contained biodevices for continuous monitoring of different bioanalytes in a human body. A future application for EFCs could also be to not only utilise EFCs as pure power sources, but also their integration with sensing systems to provide a trigger mechanism as switchable EFCs. The concept of inhibition- and activation-based biosensors has attracted a large interest recently, with *e.g*. pH switchable [52], antigen-antibody [53] and DNAzyme systems [54], as well as using the EFC directly as a glucose-sensing device [55] being reported in the literature.

For the sake of demonstration of the proof-of-principle use of actual biological fluids was avoided. However, operation of CDH based bioanodes and Lc based biocathodes have been investigated previously under physiological conditions including human fluids, such as blood, plasma, serum, and saliva [7], [32], [50], [56]–[58]. It was shown that in simple air saturated buffer solutions, bioanodes usually limit the performances of EFCs, whereas under implant conditions the limiting electrode is not always as clear. Both biooxidant and biofuel are available at limited amounts in biological fluids *in vivo* (*cf.* 0.25 mM oxygen in air saturated solution to ∼0.05 mM in blood and ∼5 mM glucose in blood) [48] and slow diffusion can severely reduce the biodevice performance. Although many problems exist in the design of efficient EFCs for operation *in vivo*, the topic has attracted increasing interest, with several reports of actually implanted devices appearing, as summarised in a recent review [7]. Different strategies can be employed to increase the performance of the devices, such as ensuring a good enzyme coverage on the bioelectrode surface by covalent immobilisation to reduce the effect of interfering compounds and allowing for optimal orientation of the enzyme (as employed for the biocathodes utilised here for the described EFC), and utilising co-immobilization strategies to increase the coulombic efficiency attained by the bioanodes [38]–[40], [59]–[61].

## Conclusions

Self-contained bioelectronic devices hold promise and substantial investment has been devoted to resolving the conflict between the required input parameters and device performance, *i.e.*, voltage and power required versus appropriate scaling, to operate a potentially implantable biodevice. This issue, amongst others, at present hampers the practical realisation of such devices. The largest component in contemporary commercially available implantable devices is the power source, and either needs to be replaced, as with batteries, or supplied with energy through electromagnetic radiation, as with RF powered devices. By employing an EFC as a power source, as described herein, power generation can be achieved autonomously with possibilities for miniaturisation even down to nm scale. By developing electronic platforms and biosensors, and designing a multi-enzyme FC, we have been able to demonstrate wireless radio signal monitoring of lactose and oxygen using only the EFC as energy source, providing enough power to closely match the designed electronics components. Employing an EFC allowed the realisation of self-powered wireless biosensing devices. Practically, this self-powered biosensing system could be combined with any other biosensors of interest, monitoring other biomolecules of interest.

The main goal of this paper is to demonstrate the proof-of-principle of wireless biodevices based on radio signal transmission, which is supplied with electrical energy by an EFC. The technology to miniaturise the electronic components is becoming increasingly available. Several strategies can be employed, and are under investigation by the research consortium to further improve the power source technology by, for instance, increasing power extraction efficiency and power output stability whilst reducing overall EFC dimensions. This type of autonomous biodevice will doubtlessly play an important role in the future, not only in medical devices, but also in applications in high-tech industry, environmental monitoring, and biocomputing.

## Supporting Information

Figure S1 **Charge pump.** Schematics of the charge pump power supply.
(TIF)

Figure S2 **Radio prototype.** Photograph of the radio prototype with folded 2.45GHz antenna.
(TIF)

Figure S3 **USB receiver.** Receiver radio with USB connector for easy connection to PC terminal.
(TIF)

Figure S4 **Low current measurement.** Schematic of an electrical circuit for measuring low current. The circuit consists of two operational amplifiers, O1 and O2, and has a sensitivity regulating input resistor, R1 (1 MΩ). The glucose or oxygen sensitive sensor was connected to input B1 and through the constant load resistor, R33 (shown in Fig. S1), and input B2 to one of the electrode of biofuel cells (biocathode or bioanode, respectively).
(TIF)

Figure S5 **Radio module operation.** The radio module is powered on by the energy harvesting module, samples the data, and transmit the information all within 4.4 ms. The controlled voltage for the radio is shown in magenta, the voltage just before the regulator in blue, dropping as the radio draws current, and the current consumption in green.
(TIF)

Figure S6 **PC software.** Photograph of the PC software taken at one of the measurements of the oxygen concentration in a remote initially air-saturated solution containing the sensor unit, receiving measured test data. The figure shows in turn the signal from unsaturated (1) and saturated (2) electronics, as we as sensor signal from air (3) and oxygen (4) saturated solutions.
(TIF)

Figure S7 **Carbohydrate sensing calibration.** Response unit from the preliminary tests of the wireless self-powered device for sugar monitoring. The dashed line represents the background signal from the electronics.
(TIF)

Figure S8 **Oxygen sensing calibration.** Response unit from the test of the wireless self-powered device for oxygen monitoring. The dashed line represents the background signal from the electronics.
(TIF)

Table S1 **Electrode characteristics.** Electrode performance, from order of appearance in the Materials section in the manuscript.
(TIF)

Text S1 **Supporting Information Text.**
(DOC)
