# Supplementary figures and images for: Self-Powered Wireless Carbohydrate/Oxygen Sensitive Biodevice Based on Radio Signal Transmission
Source: PLoS One. 2014 Oct 13;9(10):e109104. doi: 10.1371/journal.pone.0109104 (PMC4195609; doi:10.1371/journal.pone.0109104)

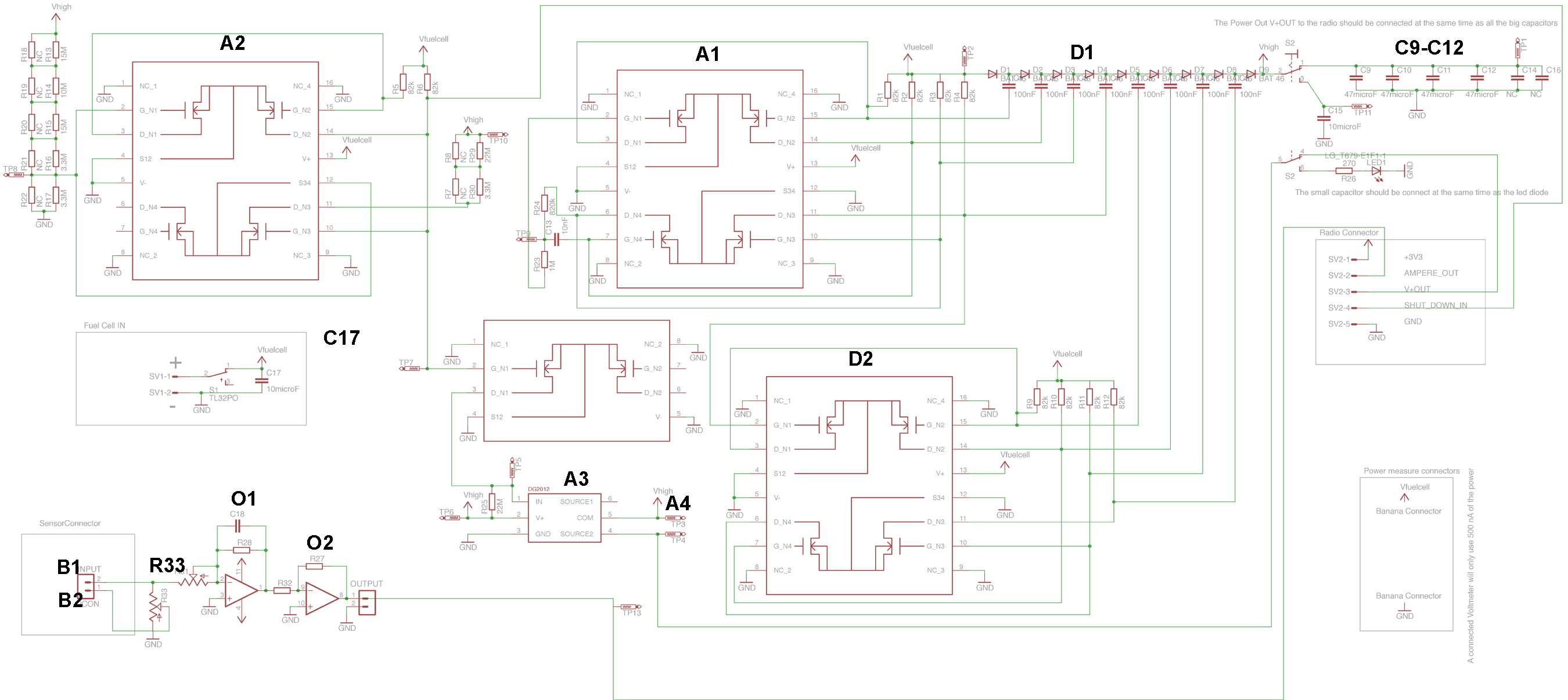

Supplement: Figure S1 — Charge pump. Schematics of the charge pump power supply. (TIF) [file pone.0109104.s001.tif]

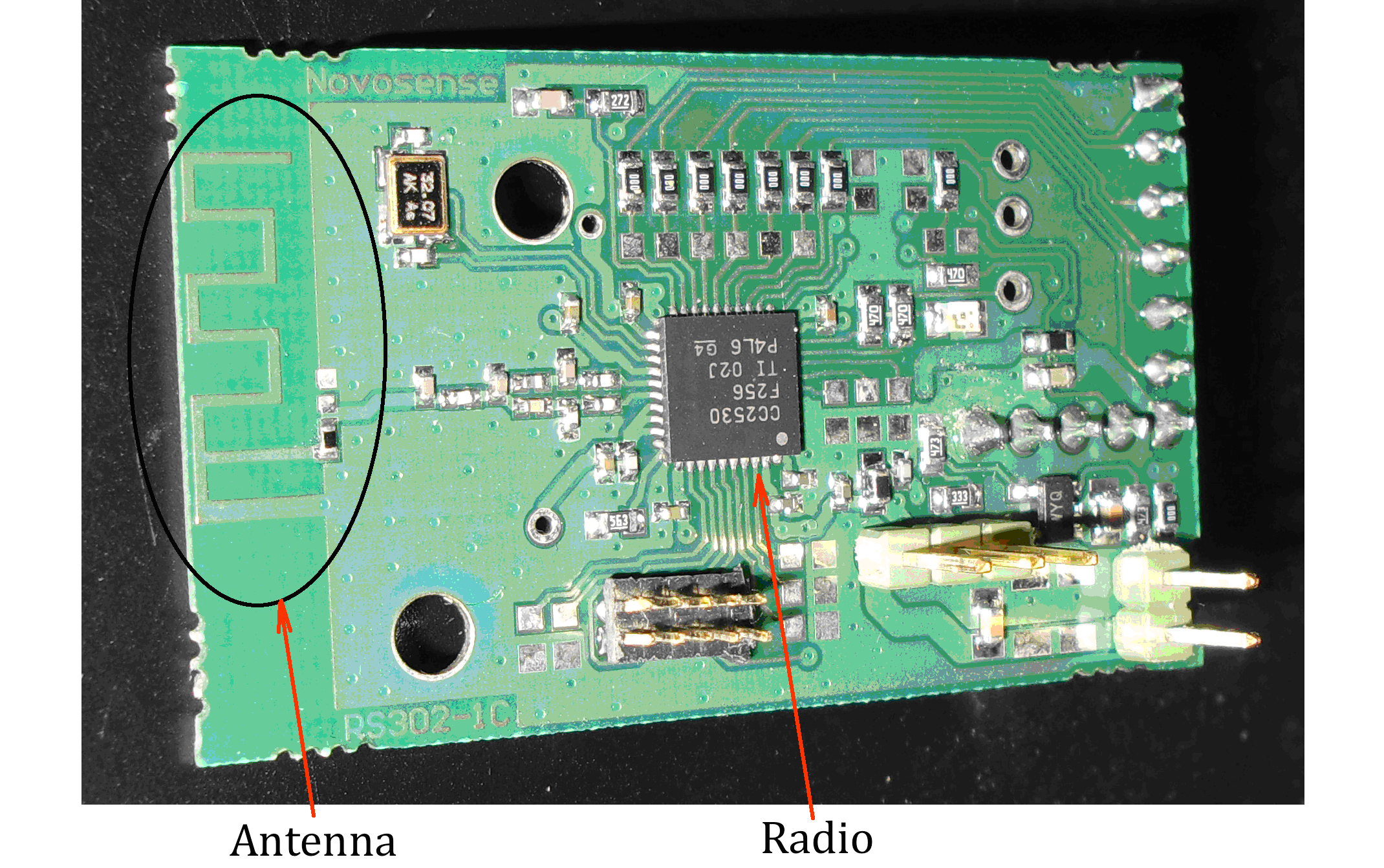

Supplement: Figure S2 — Radio prototype. Photograph of the radio prototype with folded 2.45GHz antenna. (TIF) [file pone.0109104.s002.tif]

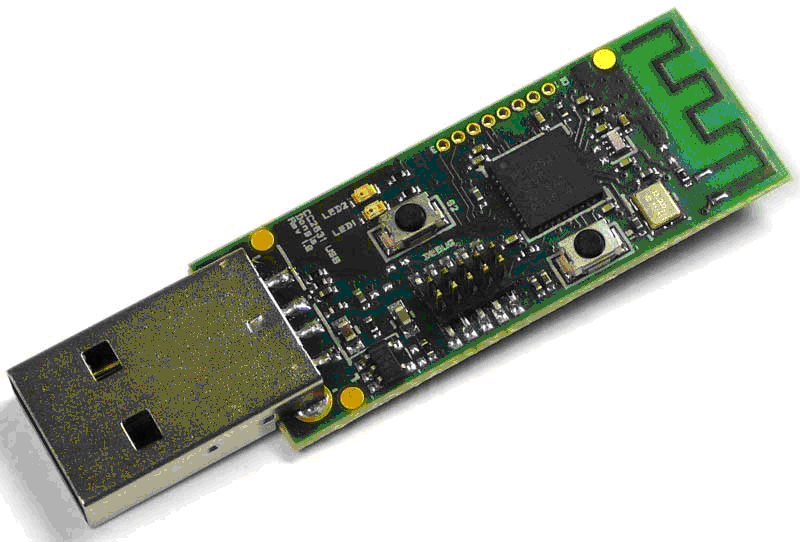

Supplement: Figure S3 — USB receiver. Receiver radio with USB connector for easy connection to PC terminal. (TIF) [file pone.0109104.s003.tif]

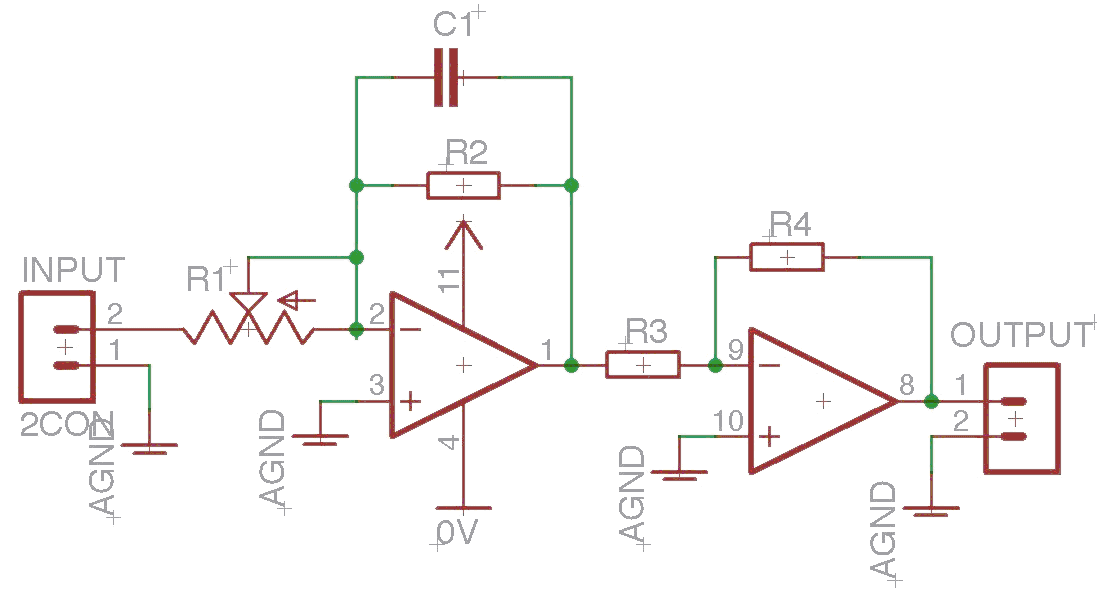

Supplement: Figure S4 — Low current measurement. Schematic of an electrical circuit for measuring low current. The circuit consists of two operational amplifiers, O1 and O2, and has a sensitivity regulating input resistor, R1 (1 MΩ). The glucose or oxygen sensitive sensor was connected to input B1 and through the constant load resistor, R33 (shown in Fig. S1), and input B2 to one of the electrode of biofuel cells (biocathode or bioanode, respectively). (TIF) [file pone.0109104.s004.tif]

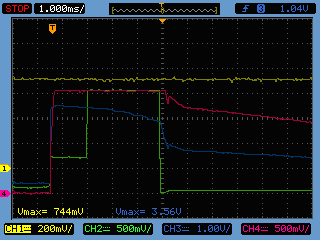

Supplement: Figure S5 — Radio module operation. The radio module is powered on by the energy harvesting module, samples the data, and transmit the information all within 4.4 ms. The controlled voltage for the radio is shown in magenta, the voltage just before the regulator in blue, dropping as the radio draws current, and the current consumption in green. (TIF) [file pone.0109104.s005.tif]

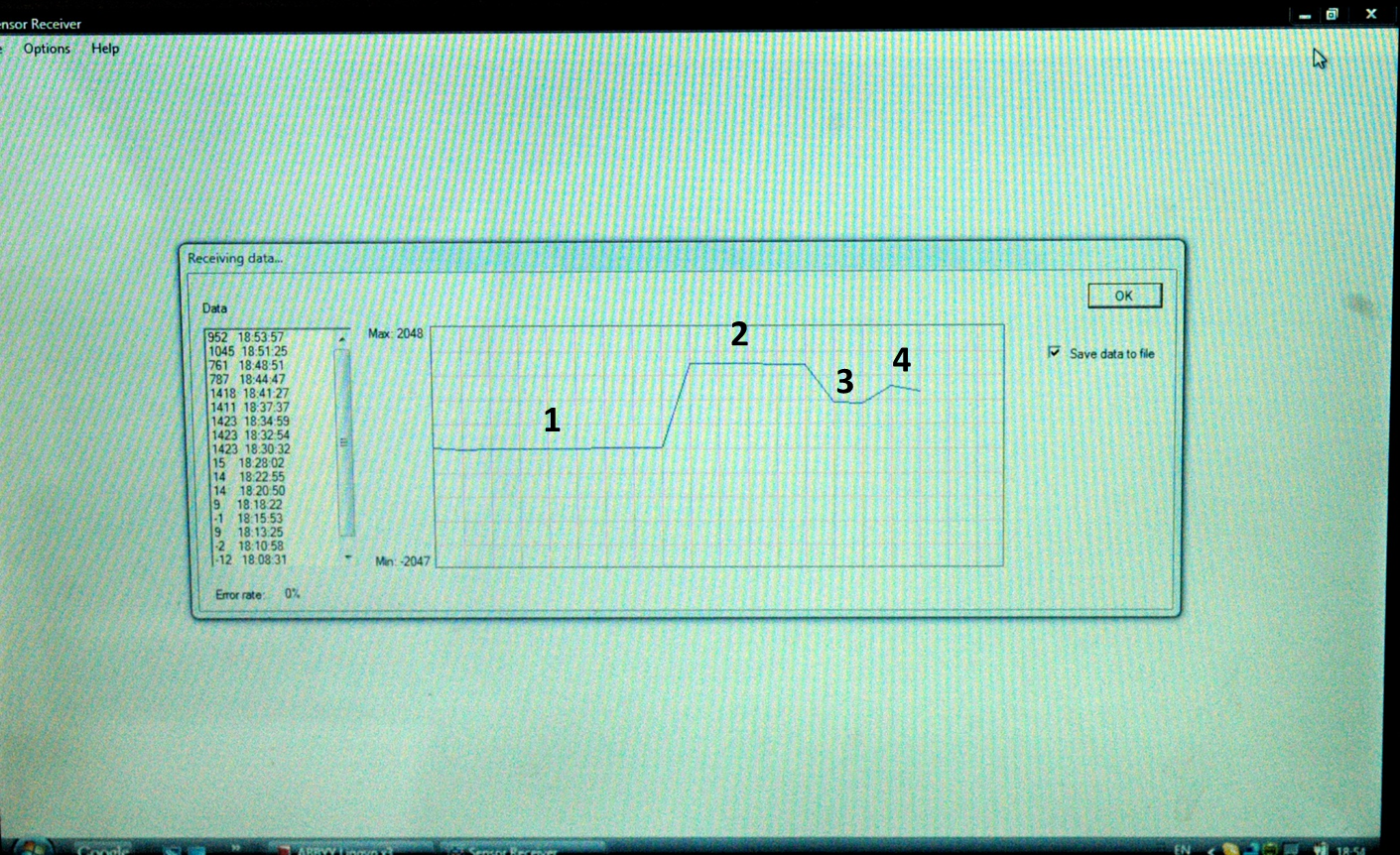

Supplement: Figure S6 — PC software. Photograph of the PC software taken at one of the measurements of the oxygen concentration in a remote initially air-saturated solution containing the sensor unit, receiving measured test data. The figure shows in turn the signal from unsaturated (1) and saturated (2) electronics, as we as sensor signal from air (3) and oxygen (4) saturated solutions. (TIF) [file pone.0109104.s006.tif]

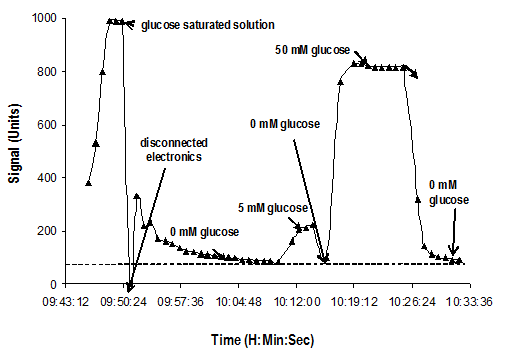

Supplement: Figure S7 — Carbohydrate sensing calibration. Response unit from the preliminary tests of the wireless self-powered device for sugar monitoring. The dashed line represents the background signal from the electronics. (TIF) [file pone.0109104.s007.tif]

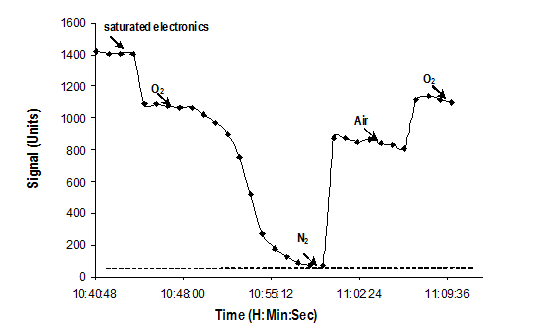

Supplement: Figure S8 — Oxygen sensing calibration. Response unit from the test of the wireless self-powered device for oxygen monitoring. The dashed line represents the background signal from the electronics. (TIF) [file pone.0109104.s008.tif]

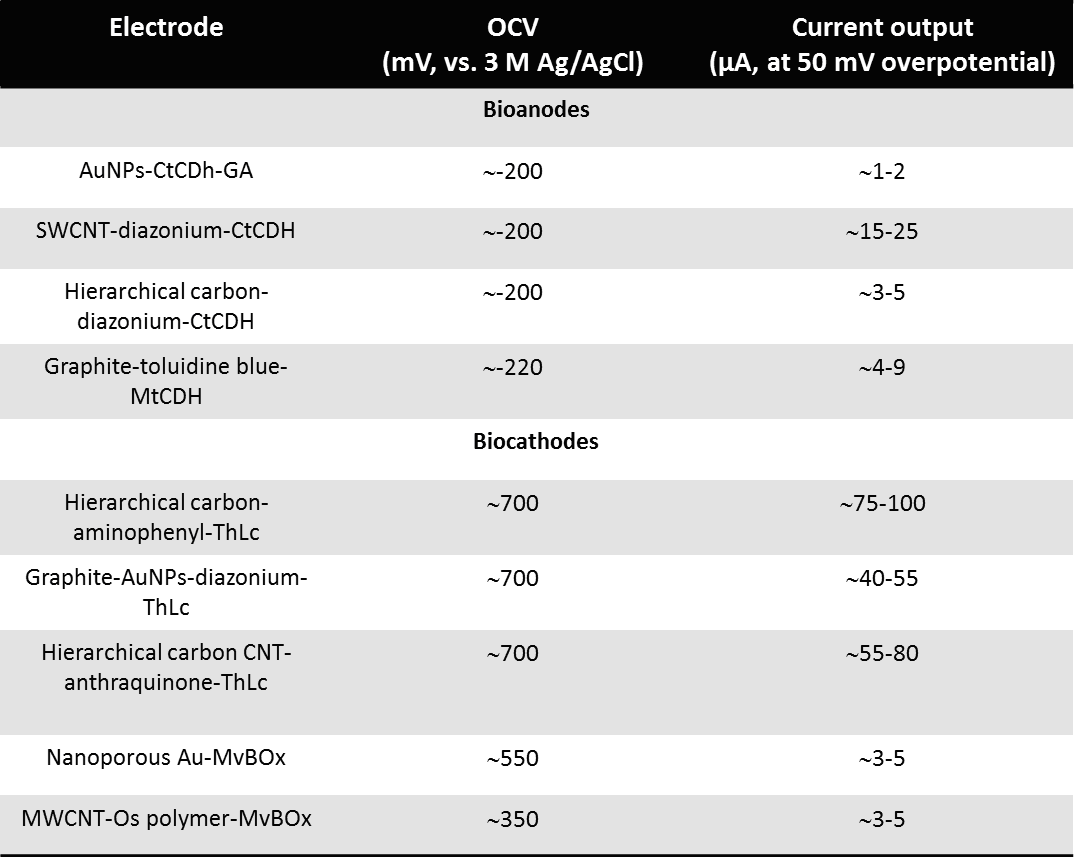

Supplement: Table S1 — Electrode characteristics. Electrode performance, from order of appearance in the Materials section in the manuscript. (TIF) [file pone.0109104.s009.tif]
