# Supplementary material for: Self-Powered Wireless Carbohydrate/Oxygen Sensitive Biodevice Based on Radio Signal Transmission
Source: PLoS One. 2014 Oct 13;9(10):e109104. doi: 10.1371/journal.pone.0109104 (PMC4195609; doi:10.1371/journal.pone.0109104)
Supplement: Text S1 — Supporting Information Text. (DOC) [file pone.0109104.s010.doc]

**Self-powered wireless carbohydrate/oxygen sensitive biodevice based on radio signal transmission**

Magnus Falk^a^, Miguel Alcalde^b^, Philip N. Bartlett^c^, Antonio L. De Lacey^b^, Lo Gorton^d^, Cristina Gutierrez-Sanchez^b^, Raoudha Haddad^e^, Jeremy Kilburn^f^, Dónal Leech^g^, Roland Ludwig^h^, Edmond Magner^i^, Diana M. Mate^b^, Peter Ó Conghaile^g^, Roberto Ortiz^d^, Marcos Pita^b^, Sascha Pöller^e^, Tautgirdas Ruzgas^a^, Urszula Salaj-Kosla^i^, Wolfgang Schuhmann^e^, Fredrik Sebelius^j^, Minling Shao^e^, Leonard Stoica^e^, Cristoph Sygmund^g^, Jonas Tilly^j^, Miguel D. Toscano^k^, Jeevanthi Vivekananthan^e^, Emma Wright^c^ and Sergey Shleev*^a^

*^a^Biomedical Sciences, Faculty of Health and Society, Malmö University, Malmö, Sweden*

*^b^Institute of Catalysis and Petrochemistry, Madrid, Spain*

*^c^Chemistry, Faculty of Natural and Environmental Sciences, University of Southampton, Southampton, UK*

*^d^Analytical Chemistry/Biochemistry and Structural Biology, Lund University, Lund, Sweden*

*^e^Analytische Chemie, Ruhr-Universität Bochum, Bochum, Germany*

*^f^School of Biological and Chemical Sciences, University of London, London, UK*

*^g^School of Chemistry, National University of Ireland Galway, Galway, Ireland*

*^h^Food Science & Technology, BOKU-University of Natural Resources and Life Sciences, Vienna, Austria*

*^i^Chemical and Environmental Sciences, University of Limerick, Limerick, Ireland*

*^j^Novosense AB, Lund, Sweden*

*^k^Novozymes A/S, Bagsværd, Denmark*

**Supporting Information**

**Design of electronic components and PC software**

**Power supply unit**

The schematics of the power supply unit are shown in Supporting Fig. S1. Everything could be implemented in silicon, except for the capacitors, which would be a minor circuit implementation.

## Radio Prototype

A Zigbee radio, CC2530 that uses the 2.4 GHz radio band, was utilized in the design combined with a folded PCB antenna, approximately 3 cm, as shown in Fig. S2. The prototype was manufactured to test different electrical setups and therefore a lot more signals and pins were attached to the radio module than later needed. Future modules can therefore be made much smaller.

Testing software for the sensor providing continuous radio transmission, as well as analysis software providing statistics for the radio link were implemented. The packet error for radio is fairly low until the critical radio signal level is reached; thereafter substantial packet loss is measured. The measurements were performed in a typical office environment, where interfering radio devices such as multiple WiFi networks and mobile phone Bluetooth devices were present to provide a realistic scenario. The radio together with the antenna was encapsulated using a thin layer of plastics and submerged in water with a level of 0.9% saline, where the packet error ratio was as low as 1% (basically the same as air) and had a range of 5 m, which was the maximum as to the restriction of the setup. The distance between the water surface and antenna during our simplified tests was approximately 10 cm mimicking an implanted situation.

A small radio prototype was used to evaluate the wireless transmission system setup, Supplementary Fig. S3. This prototype has an integrated antenna and a USB interface. It can be connected to a PC and serves as the receiver radio unit.

## Embedded software control

To minimize the power used by the radio, a protocol was developed to have the radio module completely turned off as much as possible. Specifically, when the voltage at the high capacitors (C9-C12, Fig. S1) has reached 3.8 V, the high performance switch (A3, Fig. S1) turned the radio and the sensor measurement circuit (O1 and O2, Fig. S1) on. After the transmission of the information packet the radio turned the high performance switch off, thus, terminating the power supply to the radio and the sensor measurements circuit. The whole measurement and transmission procedure had a duration of 4.4 ms, as detailed in Supplementary Fig. S5.

At maximum, the current consumption reaches roughly 45 μA, corresponding to when the radio is transmitting. As stated in the data sheet information of CC2530, which can be downloaded for free from Texas Instruments webpage ([www.ti.com](http://www.ti.com/)), the transmitting current is 30-40 mA. Taking into account the transmitting time of 4.4 ms the required charge for the radio is 0.18 nC. The usual frequency of package transmition was ca. 1 min (*vide infra*). It means that total consumption for transmitting one package was ca. 2.7 nC, which is 15 times higher. Directly after the radio has transmitted the measured value it signals the energy-harvesting module to switch off power, shown in the drop of the green curve. The reason for the rapid drop in the two voltages is the local compensators around the radio, which are implemented for decoupling purposes.

## PC software

A PC software was developed to receive, log, and draw the measured signal, as shown in the photograph in Supplementary Fig. S6. The software records the time and measured signal, while the receiver is connected to the PCs USB interface. The receiver is programmed to act as a serial communication device and thus the PC software can access the receiver data. The receiver software can be operated in the background and the sampling frequency can vary as the receiver software is in receiving mode at all times.

**Calibration of electronics**

The carbohydrate and oxygen sensing units were tested and calibrated prior to assembly of the whole biodevice using a regular low voltage battery as power source. Microscale carbohydrate and oxygen sensitive electrodes were employed, designed as described in detail previously (utilizing the cathode as oxygen sensing element and the anode as carbohydrate sensing element).^1^

For calibration of the carbohydrate sensitive unit, glucose was chosen. The anodic bioelement is sensitive to several different carbohydrates, such as glucose, cellobiose, and lactose. The glucose concentration in the electrochemical cell, with a total volume of 10 ml, was changed, and the wireless signals corresponding to different concentrations were recorded with results shown in Supporting Fig. S8. The parameters of two adjustable resistors were changed in accordance with the concentration of the substrate in the electrochemical cell, starting with the solution of the highest concentration and ending with the solution of the lowest concentration. Specifically, when glucose saturated solution was used, the signal was adjusted to its highest value, however avoiding saturation of electronics (just below 1000 units, Supporting Fig. S7). As expected, when the electronics were disconnected, the signal dropped to 0. The final calibration test was done in the absence of substrate, but with electronics connected to measure the background electronic signal, which was equal to 84 units, *i.e.* less than 9% of the saturated signal.

Following the calibration step, the biodevice system was investigated in physiologically relevant glucose concentrations (Supporting Fig. S7). Addition of 5 mM glucose to the electrochemical cell resulted in a wirelessly measured signal of *ca.* 220 units, *i.e.* almost three times higher compared to the background value. When new solution without glucose was used again, a background signal of 90 units was registered. When the highest possible physiological glucose concentration was introduced to the cell (50 mM glucose^2^), a high signal was registered equal to 820 units. Importantly, even in this emergency case (such a high glucose concentration is very unlikely under physiological conditions) the electronics were still unsaturated.

Calibration test of the oxygen sensitive device was performed similarly as the carbohydrate sensitive device. First, the procedure to calibrate the system was developed. The parameters of two adjustable resistors were changed in accordance with the concentration of substrate in the electrochemical cell, with the total volume of 10 ml, starting with an oxygen saturated solution and ending with a nitrogen saturated solution. Specifically, when oxygen saturated solution was used, the signal was adjusted to its highest value, however avoiding saturation of the electronics (above 1000 but below 1400 units, Supporting Fig. S8). The second calibration point was taken in the absence of substrate, but with the electronics connected to measure the background signal, which was close to 70 units, *i.e.* less than 7% of the highest signal (Supporting Fig. S8). Following the calibration, the oxygen concentration in the electrochemical cell was increased from 0 mM (N_2_ saturated solution) to 0.25 mM (air saturated solution), whereupon the signal increased from *ca.* 70 units (background signal from the electronics) up to 870 units (Supporting Fig. S8). Subsequent addition of oxygen into the system (O_2_ saturated solution with *ca.* 1.2 mM O_2_ concentration) resulted in a signal close to 1100, *i.e.* an almost identical value to that obtained at the beginning of the test.

To conclude, the developed carbohydrate and oxygen sensing electronic units demonstrated stable and reliable wirelessly monitored signals.

**Performance of bioelectrodes**

The actual current output and OCV of the bioelectrodes described in the Materials sections are shown in Supplementary Table S1 below. A detailed characterisation of the performance of the individual bioelectrodes can be found in the respective references, where the bioelectrodes were first described.

# Supporting References

# 1. Falk M, Andoralov V, Blum Z, Sotres J, Suyatin DB, Ruzgas T, Arnebrant T, Shleev S (2012) Biosen Bioelectron 37: 38-45.

# 2. Guyton AC and Hall JE (2006) Textbook of medical physiology. Philadelphia: Elsevier Saunders. 1116 p.
